# Fertility-preserving local excision under a hysteroscope with combined chemotherapy in a 6-year-old child with clear cell adenocarcinoma of the cervix: A case report and review of the literature

**DOI:** 10.1097/MD.0000000000018646

**Published:** 2020-01-31

**Authors:** Yuehui Su, Chunyan Zhang, Wenjing Hou, Yuligh Liou, Yueyue Chen, Ya Xie, Dongya Zhang, Pengcheng Ji, Renyin Chen, Guozhong Jiang, Mengzhen Zhang

**Affiliations:** a Department of Gynecology, First Affiliated Hospital of Zhengzhou University, Henan; b Hunan Key Laboratory of Pharmacogenetics, Institute of Clinical Pharmacology, Central South University, Changsha; c Department of Pathology, First Affiliated Hospital of Zhengzhou University, Henan, P. R. China.

**Keywords:** case report, chemotherapy, clear cell adenocarcinoma of the cervix, PAX1 methylated gene

## Abstract

**Introduction:**

Clear cell adenocarcinoma of the cervix (CCAC), a rare and more severe type of gynecological cancer, is especially rare in pediatric patients. Traditionally, surgery following chemotherapy (CT) and radiation therapy is the preferred treatment for CCAC; however, patients have poor 5-year survival rates than other types of cervical cancers.

**Patient concerns:**

A 6-year-old girl with a history of vaginal discharge for 18 months was diagnosed with CCAC by histological examination. Her parents refused the traditional treatment of radical hysterectomy and lymph node dissection because of her young age.

**Diagnosis:**

The patient's tests revealed negative human papilloma virus and negative methylated paired box 1 gene results. The tumor mass histopathology revealed stage IIA1 CCAC that originated from the cervix.

**Interventions:**

Tumor mass excision with preservation of the cervix by electrosurgical biopsy under hysteroscopy was performed. Four cycles of docetaxel and oxaliplatin CT were administered every 3 weeks.

**Outcomes:**

No signs of recurrence were observed in the 28 months after final treatment and diagnosis on magnetic resonance imaging, color ultrasonic imaging, and gynecological examination. Serologic tumor biomarkers were also within normal ranges.

**Conclusions:**

This is the first reported CCAC case in which the primary treatment included electrosurgical biopsy of the polypoid mass under hysteroscopy, followed by CT without traditional treatment: radical surgery with pelvic and/or lymphadenectomy for fertility preservation. This is a new treatment approach for young CCAC patients without the use of surgery.

## 1 Introduction

Cervical cancer, ranked fourth for both incidence and mortality in carcinomas among women, had an estimated 570,000 cases and 311,000 deaths worldwide in 2018.^[1]^ In China, an estimated 98,900 new cases were reported in 2015, with 53,200 and 45,700 cases in urban and rural areas, respectively.^[2]^ Although cervical cancer remains a more severe cancer in adult women, it is rare in the pediatric subgroup, that is, population aged ≤21 years as defined by the United States of America (USA) Food and Drug Administration.^[3,4]^

In present, clear cell adenocarcinoma of the cervix (CCAC) cases accounted for approximately 4% to 9% of the cases of adenocarcinoma (AC) of the cervix.^[5]^ CCAC is reportedly associated with a history of prenatal diethylstilbestrol (DES) exposure and resulting adverse pregnancy outcomes.^[6]^ The high risk of CCAC was reported in several countries from 1947 to 1971 during which the risk of CCAC increased with age from 15 to 29 years (born after the DES exposure period), to a peak in those aged 25 to 29 years in the USA.^[5,7]^

Traditionally, surgery is the definitive treatment for CCAC in the early stages.^[8]^ The standard management of patients with early-stage cervical cancer (International Federation of Obstetricians and Gynecologists classification (FIGO) stage IA-IB1) 000251659264 is radical hysterectomy (RH) and lymph node dissection (LND) and/or radiation with or without chemotherapy (CT), even in the pediatric population.^[9–12]^ Radical abdominal trachelectomy (RAT) and partial trachelectomy (PT) were reported as a fertility-sparing approach in young women with CCAC.^[13,14]^

We report the first, very rare case of CCAC in a Chinese 6-year old girl with no prior maternal DES exposure. To the best of our knowledge, this is the first report on the use of CT in the youngest case of cervical CCAC reported worldwide, without RH or RAT. To preserve the uterus, the tumor was resected using hysteroscopy, followed by 4 cycles of CT. The girl continues to be healthy, with no recurrence or side effects at 28 months posttreatment.

## 2 Case report

### 2.1 Patient history and examination

Informed written consent was obtained from the patient's legal guardians for publication of this case report and accompanying images. The child had a history of abnormal vaginal discharge for 3 months at the age of 5 years. She was diagnosed with a urinary tract infection by a pediatrician and treated accordingly for 3 months. Her symptoms improved during that time. Eighteen months later, she visited a gynecologist because of continuous vaginal discharge, and examination revealed a small amount of bloody discharge at the vaginal orifice. A polypoid mass, with irregular nodule texture, was detected in the upper part of the vagina during anal digital examination.

### 2.2 Image results

**Figure 1:**
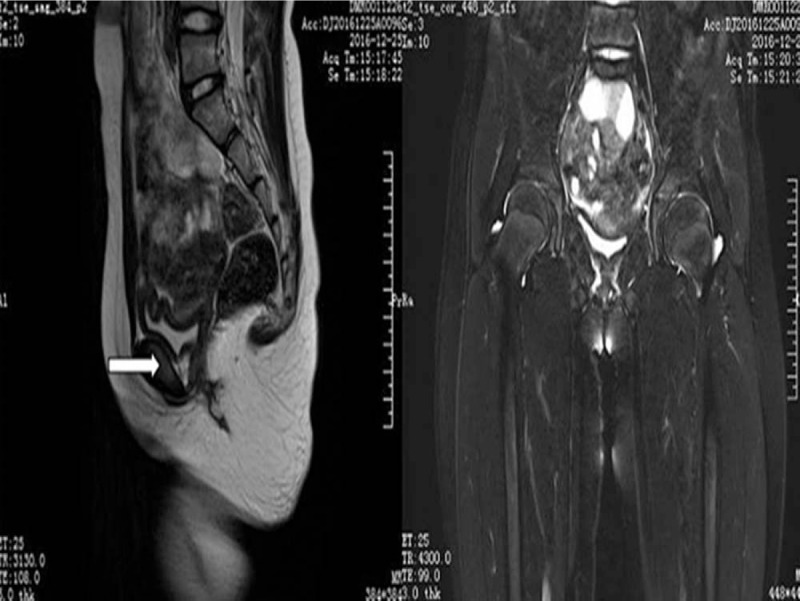
T2-weighted contrast-enhanced magnetic resonance imaging (MRI) results: preoperative (axial): a cystic lesion with a solid component was detected in the uterine cervix (arrow). An estimated measurement of 11mm × 16mm × 13 mm solid occupation can be seen at the cervix. The tumor mass was arising from the cervix into the vagina without breaking through the serous layer. preoperative (coronal): a highly suspicious solid malignant lesion occupying the tip of the cervix. MRI = magnetic resonance imaging.

Magnetic resonance imaging (MRI) revealed an 11 × 16 × 13 mm tumor mass arising from the cervix into the vagina, without breaking through the serous layer (Fig. 1A and 1B). The fornix of the vagina was not clear. There was no evidence of enlarged lymph nodes bilaterally in the iliac vessels or in the inguinal region (all were less than 10 mm).

**Figure 2:**
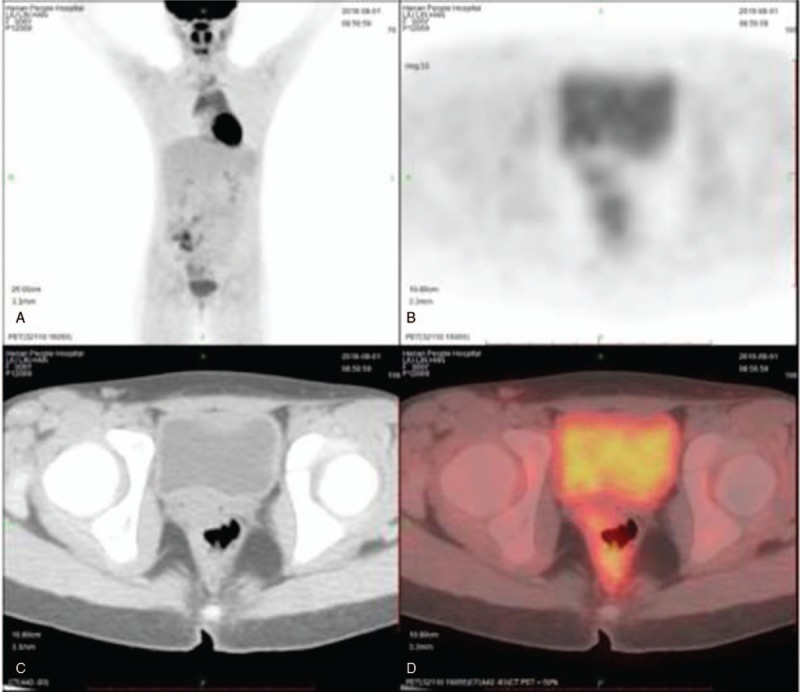
Positron emission tomography-computed tomography (PET-CT): preoperative (axial): the whole-body image scan with no other suspicious heterogeneously enhanced masses in the lymph nodes or any other organ. preoperative (coronal): a well-defined heterogeneously enhanced mass (metabolic map) at the tip of the right lateral wall of the vagina. preoperative (coronal): the lower abdomen and the cervix-vaginal area of the CT image. preoperative (coronal): The metabolic map of the same area as the Figure 2C photograph. PET-CT= positron emission tomography-computed tomography.

Positron emission tomography-computed tomography showed a well-defined heterogeneously enhanced mass at the tip of the right lateral wall of the vagina. No abnormal uptake of F-fluorodeoxyglucose was observed in the lymph nodes or in any other organ (Fig. 2).

### 2.3 Diagnosis

The result of the high-risk human papilloma virus (HPV) test using cobas 4800 HPV (Roche, Pleasanton, CA) was negative. The low methylation level of the paired box 1 (PAX1) gene was detected by a PAX1 DNA detection kit following DNA bisulfite treatment (Hoomya Inc., China). Serum tumor markers including alpha-fetoprotein (1.14 ng/mL), carbohydrate antigen-125 (CA-125; 13.17 u/mL), carbohydrate antigen 19-9 (3.96 u/mL), squamous cell carcinoma antigen (SCC-Ag; 0.754 ng/mL), carcinoembryonic antigen (CEA; 0.76 ng/mL), and human epididymal protein 4 (63.86 pmol/mL) were within normal ranges. Other biochemical parameters in the blood were also normal.

### 2.4 Treatment

**Figure 3:**
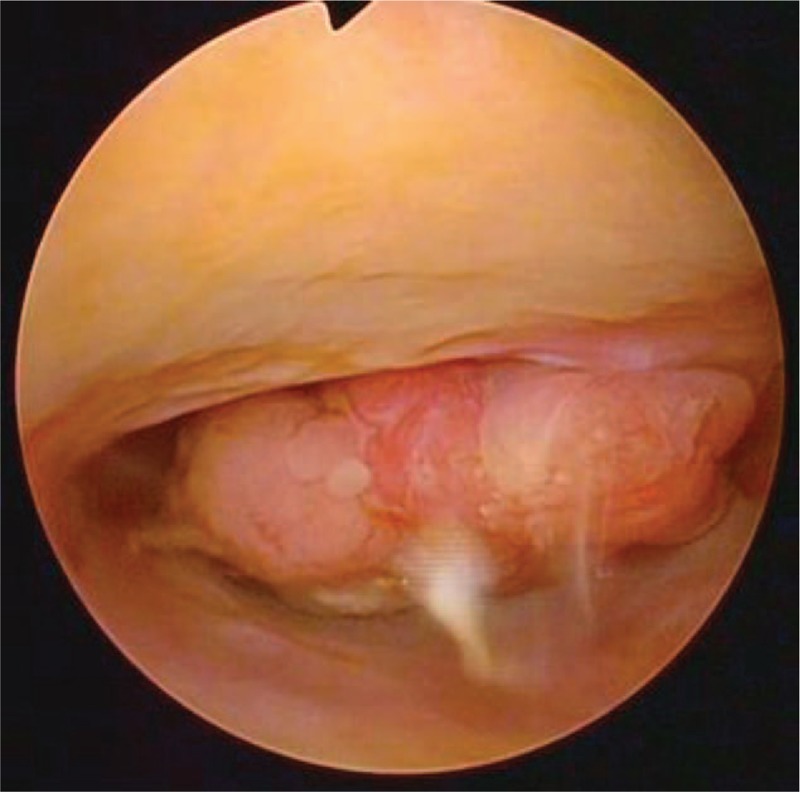
Under hysteroscopy (GE discovery VCT, GE Healthcare), a solid mass, occupying a position at about 1.5 × 2 cm in the cervix, can be seen.

Her parents refused the traditional treatment of RH and LND because of possible harm in the young age. A hysteroscopy was performed, and a 1.5 × 2 cm irregular mass was detected in the upper vagina and cervix with clear boundaries (Fig. 3). The cervix was not visualized, and the vagina fornix was shallow. Mass excision with preservation of the cervix by electrosurgical biopsy under hysteroscopy was performed. Four cycles of CT with PT (60 mg docetaxel and 100 mg oxaliplatin) were administered every 3 weeks. No adverse or unanticipated events happened during the treatment period. The patient remained disease-free at the time this manuscript was written (28 months after final treatment, end of October, 2016) according to the results of the MRI, color ultrasonic imaging, serologic tumor biomarkers, and gynecological examination.

### 2.5 Histological analysis

**Figure 4:**
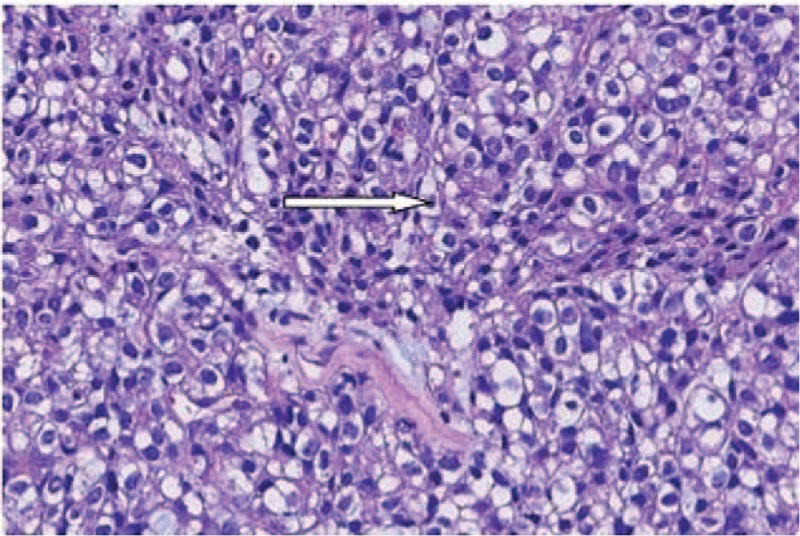
A large number of different types of cells were seen under the microscope, with bright and nested cytoplasm and with some being hollow or ring shaped.

Histological examination revealed an infiltrating tumor with a tubular-cyst pattern, clear cytoplasm, and irregular hyperchromatic nuclei of CCAC, and was confirmed by 3 pathologists (Fig. 4). On immunohistochemical examination, the tumor cells were positive for P53(20% positive), cluster of differentiation (CD)15, CK7, PAX-8, EMA, P16, HNF-1beta, and AE1/AE3. The tumor cells were negative for CD99, AX-2, and Vimentin. The patient was diagnosed with CCAC (stage IIA1), in accordance with the FIGO classification.

This study complied with the declaration of Helsinki and was approved by the Human Ethics and Research Ethics Committees of the First Affiliated Hospital of Zhengzhou University. The participating patient provided written informed consent.

## 3 Discussion

### 3.1 Types of ACs and DES exposure

Although there has been a marked decline in the incidence of cervical squamous cell carcinoma (SCC) due to screening programs and vaccination, there has been a rise in the incidence of cervical AC, especially in young women.^[15]^ ACs are histologically categorized into mucinous, endometrioid, clear cell, serous, and mesonephric subtypes, and are characterized by endocervical growth. This growth leads to the bulky enlargement of the cervix, making medical assessment difficult. CCAC is a rare disease with fewer incidences in adolescents. In the past, CCAC was linked to DES exposure in utero; nonetheless, these tumors still rarely develop despite the discontinuation of DES. CCAC is a rare type of AC. In 80% of the DES-exposed cohort of American women born between 1951 and 1956, CCAC reportedly occurred primarily among those aged between 15 and 31 years, with a small second peak occurring at around 42 years.^[16]^ In contrast, tumors in non-DES-exposed women demonstrated single peaks at ages 50.8 and 53 years, according to two different studies.^[8]^ Characteristically, CCAC may present in very young subjects, as seen in our case, due to developmental factors paralleling DES effects.^[13]^

### 3.2 Abnormal versus normal AC symptoms

Unlike most cancer symptoms in adult women, where any abnormal vaginal bleeding or contact bleeding should lead to prompt medical attention and investigations, pediatric patients usually present with vaginal bleeding and are often misdiagnosed with precocious puberty or anovulatory bleeding. Therefore, the diagnosis of ACs in children is usually difficult and delayed. Our patient was only 5 years old when she first presented with symptoms of vaginal discharge and this led to the delay of more than 18 months after her first gynecologist visit (during which the diagnosis of urinary tract infection was made). Another reason for the delay was that pelvic examination was not performed because the girl had never been sexually active. Besides, most CCAC cases are reported because of the presence of vaginal bleeding, and this is the first case reported with vaginal discharge as the primary symptom. As such, gynecologists or pediatricians must consider the possibility of malignancy when they encounter vaginal bleeding or abnormal secretions.

### 3.3 Biomarkers of ACs

CCAC, one of the histological categories of ACs, is difficult to detect because most cervical cytology results tend to be negative. HPV typing in cases of cervical cancer revealed that 87% of SCC cases worldwide were positive for HPV genotyping while only 62% of ACs were found to be HPV positive.^[17]^ The 760 cases reported as classic cervical ACs showed high HPV positivity (71.8%), while the other AC subtypes had significantly lower HPV prevalence (endometrioid, 27.3%; serous, 25%; clear cell, 20%; not otherwise specified, 13.9%; and minimal deviation, 8.3%). The traditional screening program (cytology and HPV testing) and HPV vaccination will not provide full coverage for the very small subset of classical ACs and most of the rare tumor variants such as clear cell, serous, endometrioid, and minimal deviation.

Hypermethylation of the PAX1 gene was reported to be highly associated with SCC and AC development.^[18–20]^ PAX1 showed significantly higher methylation in AC than in normal cervical tissues. The odds ratio and 95% confidence interval (CI) of high methylation levels in PAX1 for the risk of developing AC was 15.7 (95% CI, 7.0–40.6).^[20]^ The methylation level of PAX1 was not detected in this case. We cannot conclude whether the CCAC disease is associated with the PAX1 methylation gene by this single case. This study points out that CCAC may be another pathway of occurrence. The discovery of different hypermethylation genes relative to CCAC could indicate the different treatment in CCAC in the future studies.

Serum tumor markers would only appear in the blood of patients with a true malignancy; the marker would correlate with the tumor stage and treatment response. Further, these markers are easily, cheaply, and reproducibly measured. Serologic tumor markers of cervical carcinoma are used for diagnosis, prognosis, clinical management, and follow-up. There are several common cervical cancer biomarkers in clinical use; however, these are not highly specific to cervical cancer development. CA-125, CA19–9, and CEA have high positive rates in cervical AC, while SCC-Ag and cytokine 19 fragment are more associated with SCC. However, SCC-Ag, which is the best studied serum marker for SCC, has been unreliable; In this case, all cytology results, high HPV risk, and serologic tumor markers were negative.

### 3.4 Treatment strategies for SCC and AC

Currently, there is no difference in the treatment strategy between cervical SCC and AC; however, they respond very differently to treatment.^[21–23]^ The standard treatment for women with early-stage cervical cancer (IA2-IB1) remains RH with pelvic lymphadenectomy.^[24]^ The most common side effects of RH with pelvic lymphadenectomy are lower urinary tract dysfunction, sexual dysfunction, and colorectal motility disorders associated with autonomic nerve damage. Findings from multiple retrospective studies have confirmed the possibility of conservative options in selected patients who are interested in retaining their fertility,^[25–27]^ including simple hysterectomy, simple trachelectomy, and cervical conization with or without sentinel lymph node biopsy and pelvic lymph node dissection (PLND). The use of radical trachelectomy (excision of the cervix and parametria only) with LND has been proposed as a fertility-sparing technique in young women who wish to retain their fertility.^[28]^ The oncological outcomes of RH and radical trachelectomy with pelvic lymphadenectomy are similar.^[29]^ Wright et al. aimed to determine factors of parametrial tumor spread and identify the subgroup at low risk of parametrial disease.^[30]^ Low risk of parametrial invasion occurred in women with normal lymph nodes, no lymphovascular space invasion (LVSI), and tumors smaller than 2 cm. The incidence of parametrial disease in this group was only 0.4%. Frumovitz et al showed the stratified low-risk metastasis and recurrent characteristics (tumor size smaller than 2 cm and no LVSI) among women with AC, SCC, or adenosquamous carcinoma.^[31]^ In our case, the patient was observed to have low risk factors for parametrial tumor spread, and we used noninvasive (image), instead of the invasive (pathology), methods to make our risk assessment, including a lack of low risk of parametrial invasion occurred in women with normal lymph nodes and no presence LVSI. Based on our case, to determine whether a CCAC is low risk, we suggest to not only evaluate the above factors but also assess the tumor growth pattern (into the vagina without breaking through the serous layer), which is critical contributor to a low risk factor.

In a Dutch study of 88 women with CCAC, 76 (88.5%) were stage I–II.^[32]^ Surgery is the definitive treatment for CCAC in the early stages, as per the AC treatment procedure. CCAC patients without lymphatic dissemination have excellent prognosis irrespective of the use of adjuvant CT (3-year overall survival, approximately 90%).^[33]^

Neoadjuvant CT is administered to reduce tumor size and facilitate optimal tumor clearance with interval radical surgery. While SCC of the cervix is regarded as a chemo-sensitive malignancy, the sensitivity of CCAC to CT is not clearly known. A carboplatin and paclitaxel (CP) regimen used in treating advanced and recurrent endometrial cancer, including clear cell cancer, has an overall response rate of 43%. However, this regimen is well tolerated with minimal toxicity.^[34]^ For, example, Singh et al treated a 13-year-old adolescent (a stage IB1 CCAC case) with neoadjuvant CT using CP followed by a laparoscopic pelvic lymphadenectomy, vaginal radical trachelectomy, and adjuvant CT. The patient elected to conserve the uterus, therefore CP were used to reduce the tumor size, which were well tolerated with no toxicity.^[35]^ Ansari et al also reported a CCAC case in an adolescent (stage IIIA of locally advanced and node-negative CCAC) treated with chemoradiation as a treatment option.^[36]^

### 3.5 Evaluation of cervical clear cell carcinoma outcomes in related literature

Seventeen cases of clear cell carcinoma of the cervix among females in the pediatric age group (0–21 years old) without DES exposure from 2003 to 2017 are summarized in Table 1. Of the 17 listed cases, 15 papers reported that their patients were disease free for at least 6 months after treatment. All cases of stage Ia to IIIa were free of disease for at least 6 months post-treatment. In particular, the IIIa case had no recurrence 12 months after chemoradiotherapy (CRT) and CT, suggesting the possibility of successful treatment with CRT followed by CT in advanced CCAC. There were three stage Ib1 6-year-old patients, the same age as our patients, who showed no recurrence eight months after treatment. In 2 of them, RAT and PLND were performed; in the other, treatment was by PT without follow-up CT. Of the 17 cases, 15 were free of their reported diseases; however, the average follow-up period was no more than 3 years, since, as reported by Hanselaar et al, local recurrence occurred within 3 years of the initial diagnosis.^[32]^ Based on the available literature in treating early-stage cervical cancer (IA2-IB1), the recommended treatment is RH with pelvic lymphadenectomy in the presence of high-risk factors for parametrial tumor spread. However, the patient's parents declined a hysterectomy and opted for the fertility conservation option. To the best of our knowledge, this is the first reported CCAC case in which the primary treatment was by electrosurgical biopsy of the polypoid mass under hysteroscopy, followed by CT without radical surgery and with pelvic and/or lymphadenectomy. The side effects of RH or pelvic exenteration were not demonstrated in our case. This study supports the conservative treatment involving partial excision of the tumor followed by CT rather than RH, especially in pediatric patients. Further, this is the first successfully treated case of CCAC that was only treated chemotherapeutically. Our patient received concurrent docetaxel-oxaliplatin therapy only because of the low toxicity and high efficacy (in this youngest patient) of this regimen compared to other recommended treatments.

**Table 1:**
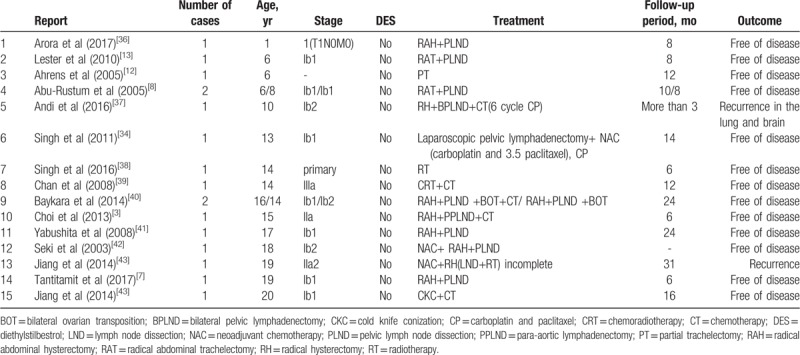
Previously reported cases of clear cell carcinoma of the cervix in pediatric patients without diethylstilbestrol exposure from 2003 to 2017.

### 3.6 Extent of fertility preservation

Hysteroscopy can assist in localization, sampling, and resection under direct vision. It provides a feasible way for young and early-stage patients with CCAC to undergo a more likely fertility-preserving surgery due to the preservation of the reproductive organs, but the long-term effect remains to be observed. The term “fertility-preservation” as used in this paper has 2 goals/meanings. The first is to reduce the reproductive organ damage caused by surgery. The second is to choose CT with less reproductive damage instead of radiotherapy. CT can kill cancer cells with a higher probability of preserving fertility. However, in our case, damage to ovarian organs due to CT drugs could not be assessed because the patient was a 6-year-old girl, so her ovarian follicles were undeveloped and still static. Static ovarian follicles are insensitive to CT, so the likelihood that fertility will be protected is maximized with this treatment.

At the latest follow-up, the patient in this study was 9 years old and healthy, but the onset of menstruation had yet to be observed. Therefore, we cannot use this case as evidence of fertility preservation. However, other retrospective studies support that CT can preserve reproductive function. In these studies, female survivors were treated with CT without radiotherapy to the pelvis or brain, given that there were few CT specific effects on fertility. In these cases, CT had a lesser effect on female fertility than on male fertility.^[44,45]^

Our study had some shortcomings. The one patient with 28 months follow-up did not allow the analysis of predictors of recurrence and the statistical power was limited. Future, this case deserves long-term follow-up and observation of possible actual fertility. The prospective studies are needed to provide more information about the safety and efficacy of this approach.

## 4 Conclusion

Although this diagnosis is rare in pediatric patients, this study strongly supports that cervical CCAC should be considered as a possible differential diagnosis of bloodstained vaginal discharge in adolescents when there is no history of sexual abuse or DES exposure. Additionally, physicians should remain cautious and attempt to act early, to improve the survival rate of CCAC patients.

## Acknowledgments

The authors acknowledge Professor Congrong Liu, pathologist of the Peking University Third Hospital, department of medicine, Peking University, for pathological consultation; Associate Professor Ruihua Wang, department of nuclear medicine, the first affiliated hospital of Zhengzhou University for general advice and support.

## Author contributions

**Conceptualization:** Yuehui Su, Mengzhen Zhang.

**Data curation:** Chunyan Zhang, Wenjing Hou, Yueyue Chen, Ya Xie, Pengcheng Ji.

**Formal analysis:** Yuligh Liou.

**Funding acquisition:** Mengzhen Zhang.

**Investigation:** Yuehui Su, Wenjing Hou, Pengcheng Ji.

**Methodology:** Wenjing Hou, Yueyue Chen, Ya Xie, Dongya Zhang.

**Project administration:** Chunyan Zhang, Mengzhen Zhang.

**Resources:** Dongya Zhang, Mengzhen Zhang.

**Supervision:** Renyin Chen, Guozhong Jiang, Mengzhen Zhang.

**Validation:** Renyin Chen, Guozhong Jiang.

**Visualization:** Renyin Chen.

**Writing – original draft:** Yuehui Su, Chunyan Zhang, Wenjing Hou, Yuligh Liou.

**Writing – review & editing:** Yuligh Liou, Mengzhen Zhang.
